# Long-term susceptible fractions in networked epidemic models and their relation to the basic reproduction number

**DOI:** 10.1038/s41598-025-22158-0

**Published:** 2025-11-03

**Authors:** Sei Zhen Khong, Lanlan Su, Tryphon Georgiou

**Affiliations:** 1 Department of Electrical Engineering, National Sun Yat-sen University, Kaohsiung, 804201 Taiwan; 2 Department of Electrical and Electronic Engineering, University of Manchester, Manchester, M13 9PL UK; 3 Department of Mechanical and Aerospace Engineering, University of California, Irvine, 92697 USA

**Keywords:** Positive systems, Epidemic models, Convergence limits, Nonlinear feedback systems, Risk factors, Epidemiology, Applied mathematics

## Abstract

Compartmental epidemic models with dynamics that evolve over a graph network have gained considerable importance in recent years. Fundamental to these models is an important threshold known as the basic reproduction number (BRN) that aims to capture, on average, the tendency of a communicable disease to spread. In this paper, we develop two complementing frameworks that provide insights into the long term evolution of a wide range of compartmental epidemic models, including group and networked processes, exploring the positive feedback that is inherent in such models. Specifically, for the case of a group (resp. networked) process, we show that the proportion of the population that is susceptible to a disease (resp. the susceptible proportion in at least one subgroup) tends to a limit that is bounded from above by the reciprocal of the BRN of the respective model, thereby establishing that the BRN encodes critical information on the level of penetration of the disease into a subpopulation. The two substantially distinct scenarios, where the disease remains always present in the population or not, are discussed and the significance of the bound explained. To verify the validity of our conclusions, we apply the developed frameworks to examining various networked epidemic models, including a model that was recently introduced for a bi-virus process.

## Introduction

Compartmental models are often applied to the study of infectious diseases in epidemiology and have enjoyed various degrees of success^1–5^, including extensions such as fractional-order models to capture memory effects and nonlinear spread dynamics^6^. These models may be used to predict and analyze the spread of the diseases and potentially form the foundation on which public health interventional control strategies are based. Epidemic models are inherently nonlinear and can be prohibitively complex to analyze, especially when the analysis aims at more than a few compartmental features. Thus, detailed stability analysis of compartmental models in epidemiology is often restricted to the study of two or three compartments using mathematical tools such as fixed-point theorems, Lyapunov methods, and differential geometry^7–11^.

Epidemic models are naturally positive systems since their states represent portion of populations that have been affected by, or, are at risk of contracting a disease. Thereby, the theory of positive systems has been applied and already amply documented in the literature. Earlier studies typically targeted particular type of models, such as networked *susceptible-infected-susceptible* (SIS) models in^12,13^, group SIDARTHE models in^14^, and networked SAIR models in^15^. Others, including^16^, have applied the theory to more general epidemic models. In this broader context, an important merit figure, the *basic reproduction number* (BRN), has been introduced to capture the ability of the disease to spread. For a precise definition of BRN in the context of compartmental disease transmission models see^16^, and for its graphical interpretation and computation see^17,18^. Evidently, the BRN figure is of great significance in epidemiology in that a BRN that is less than unity roughly means an exponentially decaying number of infections in the population, while a larger value corresponds to the disease’s exponential growth rate.

The purpose of this paper is to revisit the concept of BRN for fairly general network-based models for epidemics, and to provide quantitative bounds on the long term development of the disease. Specifically, the paper develops a *novel* positive feedback system framework that is suited for studying the steady-state values of a broad range of group (i.e., homogeneous mixing) and networked (i.e., heterogeneous mixing) epidemic models. Importantly, we show that the reciprocal of the BRN bounds the steady-state value of at least one subpopulation that is susceptible to a communicable disease, thereby providing a mathematical justification that the BRN effectively quantifies the severity of penetration of the disease into the population. This significantly enhances the existing utility and meaning of the BRN mentioned earlier by providing a metric of the level of infection in the population.

In more detail, we examine two considerably distinct scenarios in epidemiology. The first predicates on the convergence of the susceptible population to the same limit for all initial conditions of interest. In this we are interested in the existence and size of the endemic state, where the disease is always present in the population. This scenario covers, for instance, *susceptible-infected-recovered* (SIR) models with vital birth-death dynamics, and similarly networked counterparts, *networked-SIR* (nSIR), *networked-SIS* (nSIS), and so on. The main result is a novel non-strict bound on the steady-state value of the susceptible proportion of the population in a subgroup in the network in terms of the reciprocal of the BRN. This result, first announced in our preprint^19^, has already found use in the subsequent study^20^ to justify a proposed mechanism for privacy protections in networked epidemic models. We finally note that, in this paper, we do not establish convergence in complicated epidemic models with endemic equilibria — this has been an ongoing investigation in the literature. Instead, for the models we study, we assume convergence and provide bounds on the steady-state values of some subpopulations in terms of the BRNs.

In the second scenario, we consider epidemic models whose steady-state values are affected by the initial conditions. In this case, it can be shown that there is no endemic state. In such models, the infections tend to zero over time, as in the case of nSIR processes without vital dynamics. The significance of BRN however remains, in that it provides a bound on the steady-state value of the susceptible portion of the population in at least one subgroup.

Similar to^12,13,15–18^ mentioned earlier, the results in this paper are derived based on positive systems theory^21,22^. Interestingly, the recent decade has seen many developments of positive systems theory. They include robust and scalable control of positive systems^23–28^, the Kalman–Yakubovich–Popov lemma^29,30^, as well as optimal control^31^ and stochastic control^32,33^. The rich theory of positive systems has rendered compartmental models in epidemiology amenable to analysis and control via an increasing number of powerful methods.

## Organisation of the paper

The paper is structured as follows. The next section introduces the notation used throughout and presents key preliminary results. The problem under investigation is formulated in the section titled *Structure of Epidemic Models and a Feedback System Representation*. Our main results on convergence limit bounds in positive feedback systems are developed in the section *Steady-State Analysis*. These results are then applied to the study of networked models in *Networked Epidemic Models*, which also includes several numerical examples that support the validity of our findings–among them, an example involving the competitive spread of *two viruses*, as described in^34^. The paper concludes with a summary of key insights and remarks. Detailed mathematical proofs of the main results are provided in the Supplementary Information.

## Definition and preliminaries

### Matrix theory

Denote by $\mathbb {N}$, $\mathbb {R}$, $\mathbb {R}_+$, $j\mathbb {R}$, $\mathbb {C}$, $\mathbb {C}_+$, $\bar{\mathbb {C}}_-$, and $\bar{\mathbb {C}}_+$ the natural numbers, the real numbers, the nonnegative real numbers, the imaginary axis, the complex plane, the open right-half complex plane, the closed left-half complex plane, and the closed right-half complex plane, respectively. Let $|\cdot |$ denote the 1-norm. The real and imaginary parts of $s \in \mathbb {C}$ are denoted by $\textrm{Re}(s)$ and $\textrm{Im}(s)$, respectively. The $(i, j)^{\textrm{th}}$ element of a matrix $M \in \mathbb {C}^{m \times n}$ is denoted by $m_{ij}$, and we write $M = [m_{ij}]$. Given an $M \in \mathbb {C}^{m \times n}$ (resp. $\mathbb {R}^{m \times n}$), $M^* \in \mathbb {C}^{n \times m}$ (resp. $M^T \in \mathbb {R}^{n \times m}$) denotes its complex conjugate transpose (resp. transpose). When $m = n$, denote by $\lambda (M)$ and $\rho (M)$ the spectrum and spectral radius of *M*, respectively. Denote by $\lambda _i(M)$, $i = 1, \ldots , n$ the eigenvalues of *M*. Given a vector $v \in \mathbb {C}^n$, $\textrm{diag}(v) \in \mathbb {C}^{n \times n}$ denotes the diagonal matrix whose diagonal entries are $v_1, \dots , v_n$. $I_n$ denotes the identity matrix of dimension $n \times n$, and $1_n \in \mathbb {R}^n$ the column vector of all ones. The Kronecker product is denoted by $\otimes$.

Given matrices $M, N \in \mathbb {R}^{m \times n}$, we write $M \ge N$ if $m_{ij} \ge n_{ij}$ for all *i* and *j*, $M> N$ if $M \ge N$ and $M \ne N$, and $M \gg N$ if $m_{ij}> n_{ij}$ for all *i* and *j*. *M* is called a nonnegative matrix if $M \ge 0$, and positive if $M \gg 0$. Given $v, w \in \mathbb {R}^n$, let $vw \in \mathbb {R}^n$ and $\frac{v}{w} \in \mathbb {R}^n$ be such that $(vw)_i = v_i w_i$ and $(\frac{v}{w})_i = \frac{v_i}{w_i}$, respectively, and $\log (w) \in \mathbb {R}^n$ satisfy $\log (w)_i = \log w_i$, $w_i \ne 0$ is implicitly assumed. A square $M \in \mathbb {R}^{n \times n}$ is said to be Metzler if $m_{ij} \ge 0$ for all $i \ne j$, i.e., all its off-diagonal elements are nonnegative. *M* is said to be Hurwitz if every eigenvalue of *M* has strictly negative real part, i.e., $\textrm{Re}(\lambda _i(M)) < 0$ for every $\lambda _i(M) \in \lambda (M)$.

An $M \in \mathbb {R}^{n \times n}$ is said to be *irreducible* if there exists no permutation matrix *P* such that $$PMP^{-1} = \left[ {\begin{smallmatrix} {E} & {F} \\ {0} & {G} \end{smallmatrix}}\right] ,$$ where *E* and *G* are nontrivial square matrices, i.e., they are of dimensions greater than 0. The following result from Ref. [21, Corollary 2.1.5] is important for subsequent developments.

#### Lemma 1

(i) If $0 \le M \le N$, then $\rho (M) \le \rho (N)$; (ii) If $0 \le M < N$ and $M + N$ is irreducible, then $\rho (M) < \rho (N)$.

### Graph theory

A directed graph, or digraph, is a pair $\mathscr {G}= (\mathscr {V}, \mathscr {E})$, where $\mathscr {V}= \{1, \ldots , J\}$ is the set of nodes and $\mathscr {E}\subset \mathscr {V}\times \mathscr {V}$, $\mathscr {E}= \{e_1, \ldots , e_m\}$ is the set of edges such that $e_k = (i, j) \in \mathscr {E}$ if node *i* is connected to node *j*, i.e., node *i* is a neighbour of node *j*. A graph is undirected if $(i, j) \in \mathscr {E}$ implies $(j, i) \in \mathscr {E}$. A (directed) path on $\mathscr {G}$ is an ordered set of distinct vertices $\{n_0, n_1, \ldots , n_N\}$ such that $(n_i, n_{i + 1}) \in \mathscr {E}$ for all $i \in \{0, 1, \ldots , N - 1\}$.

A digraph is said to be *strongly connected* if there is a path in each direction between each pair of nodes of the graph. Given a matrix $M \in \mathbb {R}^{n \times n}$, one may associate with it a digraph $\mathscr {G}_M$ — the graph has *n* nodes labeled $1, \ldots , n$ and there is an edge connecting node *i* to node *j* if and only if $m_{ji} \ne 0$. Then *M* is irreducible if and only if its associated graph $\mathscr {G}_M$ is strongly connected Ref. [21, Theorem 2.2.7].

### Systems theory

Let $\textbf{R}$ denote the set of proper real-rational transfer functions and $\textbf{R}\mathbf {{H}}_{\infty }$ its subset of elements having no poles in $\bar{\mathbb {C}}_+$. For a linear time-invariant (LTI) system *G*, we denote its transfer function representation by $\hat{G}$. For $\hat{G}\in \textbf{R}\mathbf {{H}}_{\infty }$, let $\Vert \hat{G}\Vert _\infty$ be its $\mathbf {{H}}_{\infty }$ norm, i.e., $\Vert \hat{G}\Vert _\infty = \sup _{\textrm{Re}(s)> 0} \bar{\sigma }(\hat{G}(s))$, where $\bar{\sigma }$ denotes the largest singular value.

A nonlinear system described by

$$\begin{aligned} \dot{x}(t)&= f(x(t), u(t)), \quad x(0) = x_0 \\ y(t)&= h(x(t), u(t)), \end{aligned}$$

where *f*(*x*, *u*) and *h*(*x*, *u*) are locally Lipschitz in (*x*, *u*), is said to be *internally positive* if $x(0) \ge 0$ and $u(t) \ge 0$ for all $t \ge 0$, then $x(t) \ge 0$ and $y(t) \ge 0$ for all $t \ge 0$.

Of particular interest are linear systems that are internally positive, and a typical such LTI system having a finite state-space realisation is the form

1 $$\begin{aligned} \begin{aligned} \dot{x}(t)&= Ax(t) + Bu(t), \quad x(0) = x_0 \\ y(t)&= Cx(t) + Du(t), \end{aligned} \end{aligned}$$

where $A \in \mathbb {R}^{n \times n}$ is Metzler, $B \in \mathbb {R}_+^{n \times m}$, $C \in \mathbb {R}_+^{p \times n}$, and $D \in \mathbb {R}_+^{p \times m}$ are nonnegative matrices; see^35^. The pair (*A*, *B*) is said to be stabilisable if there exists *F* such that $A + BF$ is Hurwitz. On the other hand, the pair (*C*, *A*) is said to be detectable if there exists *L* such that $A + LC$ is Hurwitz; see Ref. [36, Chapter 3]. Obviously, when *A* is Hurwitz, (*A*, *B*, *C*) is stabilisable and detectable. In general, when (*A*, *B*, *C*) is stabilisable and detectable, $\hat{G}(s):= C(sI - A)^{-1}B + D \in \textbf{R}\mathbf {{H}}_{\infty }$ if and only if *A* is Hurwitz. Likewise, the poles of $\hat{G}$ lie in $\bar{\mathbb {C}}_-$ if and only if $\lambda (A) \subset \bar{\mathbb {C}}_-$.

The following important result on small-gain positive systems, that is a direct consequence of Ref. [22, Theorem 3], will be used repeatedly in subsequent developments.

#### Lemma 2

Consider an internally positive LTI system described by (1) with Hurwitz *A* and $D = 0$. Given $K \ge 0$, it holds that $(I - K\hat{G})^{-1} \in \textbf{R}\mathbf {{H}}_{\infty }$ if and only if $\rho (K\hat{G}(0)) < 1$.

## Structure of epidemic models and a feedback system representation

Compartmental epidemic models are made up of a compartment of people (most often expressed as a proportion of a population) that are susceptible to diseases, and other compartments of people who have been exposed to, infected with, or recovered from a disease. These models contain nonlinear terms that capture the interaction or mixing between the susceptible and disease compartments. Take, for example, the simplest compartmental (group) epidemic model — the SIS model^37^. Its dynamics are described by

2 $$\begin{aligned} \begin{aligned}\dot{S}(t)&= -\beta S(t) I(t) + \gamma I(t) \\ \dot{I}(t)&= \beta S(t) I(t) - \gamma I(t), \end{aligned} \end{aligned}$$

where *S* and *I* denote the susceptible and infected compartments respectively. The nonlinearity of the model arises from the bilinear mixing term *S*(*t*)*I*(*t*). This model will be revisited in more detail in Section *Networked epidemic models*.

**Fig. 1:**
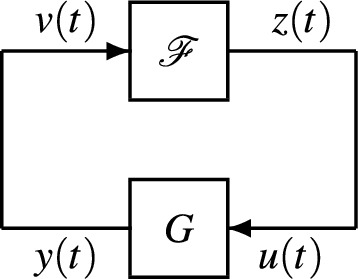
Feedback interconnection $$[G,\mathscr {F}]$$.

A crucial observation that will find use in the analysis of other epidemic models as well, is that (2) can in fact be written as the feedback interconnection of a positive nonlinear subsystem

$$\begin{aligned} \mathscr {F}: \quad {\left\{ \begin{array}{ll} \dot{S}(t) = -\beta S(t) v(t) + \gamma v(t), \quad S(0) = S_0 \\ z(t) = \beta S(t) v(t), \end{array}\right. } \end{aligned}$$

and a positive LTI system

$$\begin{aligned} G: \quad {\left\{ \begin{array}{ll} \dot{I}(t) = -\gamma I(t) + u(t), \quad I(0) = I_0 \\ y(t) = I(t), \end{array}\right. } \end{aligned}$$

in which the loop is closed by setting $v = y$ and $u = z$; see Fig. 1. Likewise, in more general epidemic models, the dynamics that capture the interaction between the susceptible compartment and other compartments, together with birth/death terms that are not directly tied to the epidemic, typically constitute the nonlinear component. Then, relaxation dynamics of the various disease compartments, such as exposed, infected, or recovered, can typically be brought together as a linear positive component of the feedback loop.

It is noteworthy that similar feedback representations may be used to describe other useful models, such as predator-prey models of the Lotka–Volterra types.

In the rest of this section we detail the structure for such a feedback system that we will employ in the paper, specifically in Section *Networked epidemic models*, which can encompass a range of epidemic models.

The type of feedback systems that we will consider, will always be thought as having the structure depicted in Fig. 1. They will consist of a nonlinear subsystem

3 $$\begin{aligned} \mathscr {F}: \quad {\left\{ \begin{array}{ll} \dot{s}(t) = f(s(t), v(t)), \quad s(0) = s_0 \\ z(t) = M_1 \left( I_d \otimes \textrm{diag}(s(t))\right) M_2 v(t), \end{array}\right. } \end{aligned}$$

where *f*(*s*, *v*) is locally Lipschitz in (*s*, *v*), $s(t) \in \mathbb {R}^{n_s}$, $M_1> 0$, $M_2> 0$ and $d\in {\mathbb {N}}$, and where further, ${\mathscr {F}}$ is assumed internally positive (by virtue of the same property of the dynamics $\dot{s}=f(s,v)$). The entries of the vector “*s*” represent susceptible portion of the population in each of a collection of networked communities, corresponding to nodes. The integer *d* represents the number of possible simultaneous infections due to different diseases. Then, the presence of a Kronecker product in the output equation allows modeling exposure of the susceptible population to multiple infections at the same time; an example of that is given in Section *Networked epidemic models*.

Next, the LTI subsystem *G* in the feedback loop will be assumed internally positive, as in (1), with state matrix *A* being Hurwitz and $D = 0$. As explained earlier, this part encompasses relaxation dynamics of the compartments in the various communities. The connection between the two subsystems corresponds to setting

4 $$\begin{aligned} v = y, \quad u = z, \end{aligned}$$

and the feedback system will be denoted by $[G, \mathscr {F}]$. Typically, in applications, it will be assumed that the initial conditions (*s*(0), *x*(0)) take values in specified regions $\mathscr {I} \subset \mathbb {R}_+^{n_s+n}$ of interest that carry specific significance. Building on these types of epidemic models, the goal of the paper is to establish metrics that quantify the level of penetration of a disease, or possibly several simultaneous diseases, in a population.

## Steady-state analysis

We now focus on the steady-state value of the susceptible states, i.e., entries of the state vector “*s*” in (3), for the system $[G,{\mathscr {F}}]$. The first subsection details our first main result, which is a bound predicated on convergence from every initial condition of interest to the same limit. The second subsection contains the second main result, which is a bound on a limit that varies with initial conditions. The next subsection gives a characterization of the limit in the second result under more restrictive assumptions on the state-space models of $\mathscr {F}$. Specializations of the derived results to epidemic models are elaborated in the final subsection.

### A bound on the susceptible population

The purpose of this subsection is to establish a bound on an entry of “*s*” asymptotically, as time goes to infinity, under the assumption that “*s*” converges to a unique equilibrium for all initial conditions in $\mathscr {I} \subset \{(s,x)\mid 0 \ll s \le 1_{n_s}, 0 \le x \le 1_n\}$.

#### Theorem 1

Consider the feedback system $[G, \mathscr {F}]$ described by (1), (3), and (4) with *A* being Hurwitz and $D = 0$. Suppose $M_2CA^{-1}BM_1$ is irreducible, and that for all initial conditions $(s(0), x(0)) \in \mathscr {I}$, $\lim _{t \rightarrow \infty } s(t) = \bar{s} \gg 0$ and $\lim _{t \rightarrow \infty } x(t) = \bar{x} \ge 0$. Then there exists $i \in \{1, \ldots , n_s\}$ such that

$$\begin{aligned} \bar{s}_i \le \frac{1}{\rho (M_2\hat{G}(0)M_1)} = \frac{1}{\rho (M_2CA^{-1}BM_1)}. \end{aligned}$$

Moreover, if $n_s = 1$, then $\bar{s} = \frac{1}{\rho (M_2\hat{G}(0)M_1)}$.

Theorem 1 makes use of nonnegative matrix theory and Lemma 2, the small-gain result for positive systems. When applied to epidemic models, Theorem 1 captures the scenario where the disease becomes endemic, i.e., $\lim _{t \rightarrow \infty } x(t) = \bar{x}> 0$, i.e., there will always be some portions of the subpopulations that carry the disease. In other words, the disease stays with the population in perpetuity.

### A bound on the susceptible population when the stationary limit varies with initial conditions

The next result, Theorem 2, shows that we may obtain a bound on an entry of the limit of “*s*” even when the irreducibility assumption fails. In this case, the limiting stationary value for the various entries may depend on the initial conditions. The bound is applicable to general multi-input-multi-output (MIMO) systems and can be seen as a generalization of Ref. [14, Proposition2]. Note that the result in Ref. [14, Proposition 2] was developed only for a specific single-input-single-output (SISO) system called the SIDARTHE model.

We begin with the following assumption, which is concerned with the function *f* in (3). Under this assumption, the limit of “*s*” may vary with the initial conditions. Let the set of initial conditions $\mathscr {I} \subset \{(s,x)\mid 0 \le s \ll 1_{n_s}, 0 \le x \le 1_n \}$.

#### Assumption 1

If $\bar{s}> 0$ and $f(\bar{s}, \bar{v}) = 0$, then $\bar{v} = 0$.

#### Theorem 2

Consider the feedback system $[G, \mathscr {F}]$ described by (1), (3), and (4) with *A* being Hurwitz and $D = 0$. Suppose that Assumption 1 holds and that for all initial conditions $(s(0), x(0)) \in \mathscr {I}$, $\bar{s}(s(0), x(0)):= \lim _{t \rightarrow \infty } s(t)$ exists and is continuous in *x*(0). Then, $\lim _{t \rightarrow \infty } x(t)=0$, and

5 $$\begin{aligned} \begin{aligned} \bar{s}_i(s(0), x(0))&< \frac{1}{\rho (M_2\hat{G}(0)M_1)} = \frac{1}{\rho (M_2CA^{-1}BM_1)} \end{aligned} \end{aligned}$$

for some $$i \in \{1, \ldots , n_s\}$$.

A part of the conclusion of Theorem 2 is that $x(t) \rightarrow 0$ as $t \rightarrow \infty$, which means in applications to epidemic models that the proportions of the subpopulations that are infected with the disease decrease to 0 asymptotically. In other words, the disease will eventually die out or be completely eradicated instead of becoming endemic. A detailed comparison between Theorem 1 and Theorem 2 is provided in Table 1. The specific applicable epidemic models will be presented in Section *Networked Epidemic Models*.

**Table 1:** Comparison between Theorem 1 and Theorem 2.

Comparison	Theorem 1	Theorem 2
Assumptions	$M_2CA^{-1}BM_1$ is irreducible, $\forall (s(0), x(0)) \in \mathscr {I}$, $\bar{s} := \displaystyle \lim _{t \rightarrow \infty } s(t)$ and $\bar{x} := \displaystyle \lim _{t \rightarrow \infty } x(t)$ uniquely exist, $\bar{s} \gg 0$ and $\bar{x} \ge 0$	$\forall (s(0), x(0)) \in \mathscr {I}$, $\bar{s}(s(0), x(0)):= \lim _{t \rightarrow \infty } s(t)$ exists, and is continuous in *x*(0); Assumption 1
Key result	$\exists i \quad \text {s. t.} \quad \bar{s}_i \le \frac{1}{\rho (M_2CA^{-1}BM_1)}$	$\exists i \quad \text {s. t.} \quad \bar{s}_i < \frac{1}{\rho (M_2CA^{-1}BM_1)}$, and, $\lim _{t\rightarrow \infty }x(t)=0$
Applicable models	Disease becomes endemic due to reinfection and/or population influx, e.g, Single-virus and Bi-virus Networked-SIS, Networked-SIRS, Networked-SIR and Networked-SEIR with vital dynamics	Disease dies out, e.g., Networked-SIR and Networked-SEIR without vital dynamics

### Characterizing the equilibria for specific models

Recall that the steady-state value of “*s*” may vary with the initial conditions in the setting of Theorem 2. The subsequent result shows that if *f* in (3) takes a specific form, the equilibrium can be characterized via a closed-form expression. The result is applicable to general MIMO systems and is a generalization of Ref. [14, Proposition 3].

#### Theorem 3

Consider the feedback system $[G, \mathscr {F}]$ described by (1), (3), and (4) with $d=1$, *A* being Hurwitz and $D = 0$. Suppose (i) Assumption 1 holds, (ii) $M_1 = I$, $f(s, v) = - \textrm{diag}(s) M_2 v$, and (iii) for all $(s(0), x(0)) \in \mathscr {I}$ such that $s(0) \gg 0$, it holds that $s(t)\gg 0,\forall t\ge 0$ and $\lim _{t \rightarrow \infty } s(t) = \bar{s} \gg 0$. Then $\bar{s}$ satisfies

$$\begin{aligned} \log \left( \textrm{diag}(s(0))^{-1}\bar{s}\right) + M_2CA^{-1} B (\bar{s} - s(0)) = M_2CA^{-1} x(0). \end{aligned}$$

Interestingly, and for a suitable choice of a matrix $M>0$, a dynamical relation $\dot{r}(t)=Mx(t)$ may be used to calculate occupation of various compartments of interest. As a corollary to the theorem and under the same assumptions, the long term behavior of such a vector *r*(*t*), starting from some $r(0) \ge 0$, can be expressed as follows

$$\begin{aligned} \lim _{t\rightarrow \infty } r(t)=:\bar{r}=r(0)+MA^{-1}(x(0) - B(\bar{s} - s(0))). \end{aligned}$$

### Applications to epidemic models

The feedback system modeled by (1), (3), and (4) can be applied to various group and networked epidemic models. This can be done by taking $s_i(t)$ as the proportion of a susceptible population and $x_i(t)$ the proportion of a population that belongs to disease compartments, where there are *N* populations in total and $i \in \{1, \ldots , N\}$. By substituting the output of (3) into (1), one obtains

$$\begin{aligned} \dot{x}(t) = Ax(t) + B M_1 \textrm{diag}(s(t)) M_2 Cx(t). \end{aligned}$$

Linearizing the model around the disease-free equilibrium ($s^* = [1, \ldots , 1]^T$, $x^* = 0$), yields

$$\begin{aligned} \dot{x}(t) = Ax(t) + B M_1M_2 Cx(t). \end{aligned}$$

Under certain assumptions Ref. [16, Theorem 2] shows that with $R_0:= \rho (BM_1M_2CA^{-1})$, the disease-free equilibrium of the epidemic model is locally asymptotically stable if $R_0 < 1$ and unstable if $R_0> 1$; see also^38^. Notice that

$$\begin{aligned} R_0 = \rho (BM_1M_2CA^{-1}) = \rho (M_2CA^{-1}BM_1), \end{aligned}$$

which is the reciprocal of the derived upper bound on the steady-state value of *s*(*t*) in Theorems 1 and 2. The value $R_0$ is of significant importance in the study of convergence to equilibria in epidemic models, and is known as the BRN mentioned earlier. It captures the average spreadability of communicable diseases and is considered a fundamental threshold in epidemiology. More specifically, it represents the expected number of secondary infections arising from an infected individual, i.e., the average number of persons to which an infected person can pass the disease.

Of particular interest is the case where $R_0> 1$, i.e., the disease-free equilibrium is unstable. Under considerably different circumstances, each of Theorem 1 and Theorem 2 provides an upper bound on an entry in $\lim _{t \rightarrow \infty } s(t)$ in the form of $\frac{1}{R_0}$. This indicates the level of penetration of the disease into at least one subpopulation. This bound becomes significant if it is much smaller than one (i.e., $R_0 \gg 1$), in which case it signifies that a large portion of the subpopulation will eventually be infected with the disease. Furthermore, in the setup of Theorem 1, *x*(*t*) generally converges to a nonzero value, which in epidemiology corresponds to the endemic state. On the contrary, when the suppositions of Theorem 2 are satisfied, it must hold that $x(t) \rightarrow 0$, meaning that there is no endemic state, i.e., the entire population that has caught the disease has either recovered or succumbed to the disease. In essence, Theorems 1 and 2 capture two distinct scenarios. In particular, Theorem 1 considers convergence from all initial conditions of interest to a unique endemic state. By contrast, Theorem 2 considers convergence to distinct steady-state values in the susceptible compartment from different initial conditions, and in this case the disease is shown to be not endemic.

## Networked epidemic models

Networked models capture the scenario where numerous groups or nodes are interconnected via a contact graph or interconnection network, defined by an adjacency matrix $W = [w_{ij}]$. Each $w_{ij} \ge 0$ quantifies the strength of the connection from node *j* to node *i*. Similarly to the group epidemic models, each group/node in a network is made up of different compartments (susceptible, infected etc.) in a networked compartmental model.

**Fig. 2:**
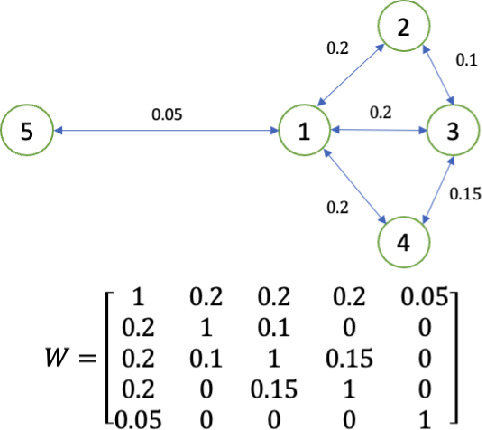
A contact graph and its adjacent matrix.

We apply the main results developed in the previous section to a range of networked epidemic models in this section. For ease of presentation, all examples in this section are simulated on the same graph (Fig. 2), which is taken to be undirected and connected. In the context of the networked models presented below, the irreducibility of the matrix $M_2CA^{-1}BM_1$ typically implies that the contact network is strongly connected, i.e., there is a path for the disease to spread from any subpopulation to any other subpopulation — the entire network is epidemiologically linked. Furthermore, where possible, we state conditions under which assumptions on the stationary limit of *s*(*t*) hold. For models for which such conditions have not been established in the literature, we note that the assumptions have been observed and verified in numerous simulations.

### Networked-SIS models

Both the standard nSIS models involving one virus and the recently introduced bi-virus nSIS models from^34^ are examined here.

#### Standard single-virus nSIS models

Consider a network of *N* nodes. Let $s_i(t)$ and $p_i(t)$ denote the proportions of population that are susceptible and infected, respectively, at time *t* and node *i*. Denote, respectively, by $\gamma _i$ and $\beta _i$ the rate of recovery from the infected population and the rate of infection due to the contact between the susceptible and infected compartments of the population, at node *i*. The entire population at every node is normalized to 1. The nSIS model^9^ is described by

$$\begin{aligned} \dot{s}_i(t)&= -\beta _i s_i(t) \sum _{j = 1}^N w_{ij} p_j(t)+\gamma _i p_i(t) \\ \dot{p}_i(t)&= \beta _i s_i(t) \sum _{j = 1}^N w_{ij} p_j(t) - \gamma _i p_i(t). \end{aligned}$$

Writing this in vectorial form $s = [s_i]$, $p = [p_i]$, $\beta = [\beta _i]$, and $\gamma = [\gamma _i]$ yields

$$\begin{aligned} \dot{s}(t)&= -\textrm{diag}(s(t)\beta ) W p(t) +\textrm{diag}(\gamma ) p(t)\\ \dot{p}(t)&= \textrm{diag}(s(t)\beta ) W p(t) - \textrm{diag}(\gamma ) p(t). \end{aligned}$$

Define the LTI system *G* as in (1) by setting

$$\begin{aligned} x(t) = p(t),\; A = - \textrm{diag}(\gamma ),\; B = I,\; C = I,\; D = 0, \end{aligned}$$

and the nonlinear component $\mathscr {F}$ as in (3), setting

$$\begin{aligned} f(s, p) = (- \textrm{diag}(s\beta )W +\textrm{diag}(\gamma ))p, \end{aligned}$$

with $M_1 = I$, $M_2 = \textrm{diag}(\beta ) W$. The nSIS model can thus be expressed as the feedback system $[G, \mathscr {F}]$. It can be verified that $M_2CA^{-1}BM_1$ is irreducible. The set of initial conditions of interest is

$$\begin{aligned} \mathscr {I} := \{(s, p) : s \gg 0, p> 0; s + p= 1_{n_s}\}. \end{aligned}$$

Suppose $s(t)\rightarrow \bar{s}\gg 0$ for all $(s(0),p(0))\in \mathscr {I}$. By applying Theorem 1, we can then conclude that for some $i\in \{1,\ldots , n_s\}$,

$$\begin{aligned} \bar{s}_i\le \frac{1}{\rho (\textrm{diag}(\beta ) W\textrm{diag}(\gamma ^{-1}))}=\frac{1}{\rho (W\textrm{diag}(\frac{\beta }{\gamma }))}=:\frac{1}{R_0}. \end{aligned}$$

##### Example 1

Consider a single-virus nSIS model over a network of 5 nodes depicted in Fig. 2.

**Fig. 3:**
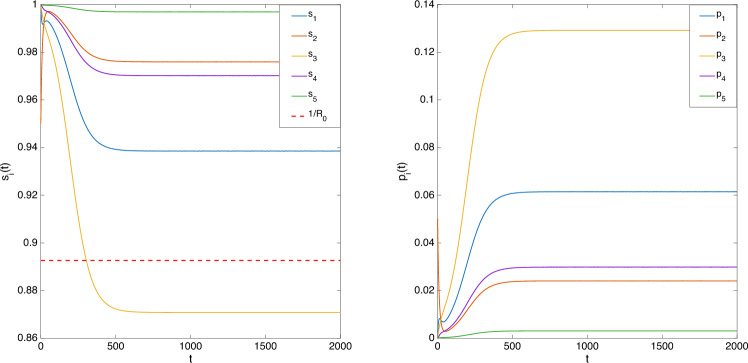
Evolution of $(s_i(t), p_i(t)),{i=1,\ldots ,5}$ in the nSIS model.

Let $\beta _1=0.2,\beta _2=\beta _3=\beta _4=0.1,\beta _5=0.05$ and $\gamma _1=0.3,\gamma _2=\gamma _4=0.2,\gamma _3=\gamma _5=0.1$. According to the preceding result, there exists *i* such that $\bar{s}_i<\frac{1}{R_0}=0.8926$. Let the initial condition be chosen from $\mathscr {I}$ as $s(0)=[1\; 0.95\; 1\;1\;1\;]^T, \; p(0)=[0\; 0.05\; 0\;0\;0\;]^T$, which simulates the scenario that the spread starts from Node 2. The evolution of (*s*(*t*), *p*(*t*)) is plotted in Fig. 3, showing that $\bar{s}_3 < \frac{1}{R_0}$.

It is noteworthy that convergence to equilibrium has been established in^9^ for a simplified nSIS model, where recovery rates $\beta _i$ and healing rates $\gamma _i$are identical across the network. To be specific Ref. [9, Theorem 4.2] shows that if $R_0>1$, then for any $(s(0),p(0))\in \mathscr {I}$, (*s*(*t*), *p*(*t*)) converges to an equilibrium $(\bar{s},\bar{p})$ that reveals an endemic state.

#### Bi-virus networked-SIS models

A bi-virus nSIS model^34^ is described by

$$\begin{aligned} \dot{s}_i(t)&= -\beta _i^1 s_i(t) \sum _{j = 1}^N w_{ij} p_j^1(t)-\beta _i^2 s_i(t) \sum _{j = 1}^N w_{ij} p_j^2(t)+\gamma _i^1 p_i^1(t)+ \gamma _i^2 p_i^2(t) \\ \dot{p}_i^1(t)&= \beta _i^1 s_i(t) \sum _{j = 1}^N w_{ij} p_j^1(t) - \gamma _i^1 p_i^1(t)\\ \dot{p}_i^2(t)&= \beta _i^2 s_i(t) \sum _{j = 1}^N w_{ij} p_j^2(t) - \gamma _i^2 p_i^2(t), \end{aligned}$$

where $s_i$, $p_i^1$ and $p_i^2$ denote the proportions of population that are susceptible, infected with virus 1 and virus 2 at the node *i*, respectively, $\gamma _i^1,\gamma _i^2>0$ and $\beta _i^1,\beta _i^2>0$ are the healing rates for virus 1 and virus 2, and infection rates for virus 1 and virus 2 at the node *i*, respectively. Writing this in vectorial form $s = [s_i]$, $p^1 = [p_i^1]$, $p^2 = [p_i^2]$, $\beta ^1 = [\beta _i^1]$, $\beta ^2 = [\beta _i^2]$, $\gamma ^1 = [\gamma _i^1]$, and $\gamma ^2 = [\gamma _i^2]$ yields

$$\begin{aligned} \dot{s}(t)&= -\textrm{diag}(s(t)\beta ^1) W p^1(t) - \textrm{diag}(s(t)\beta ^2) W p^2(t) +\textrm{diag}(\gamma ^1) p^1(t)+\textrm{diag}(\gamma ^2) p^2(t)\\ \dot{p}^1(t)&= \textrm{diag}(s(t)\beta ^1) W p^1(t) - \textrm{diag}(\gamma ^1) p^1(t)\\ \dot{p}^2(t)&= \textrm{diag}(s(t)\beta ^2) W p^2(t) - \textrm{diag}(\gamma ^2) p^2(t). \end{aligned}$$

Define LTI system *G* as in (1) by setting

$$\begin{aligned} x(t) = \left[ {\begin{smallmatrix} {p^1(t)} \\ {p^2(t)} \end{smallmatrix}}\right] , \; A = - \textrm{diag}\left( \left[ {\begin{smallmatrix} {\gamma ^1} \\ {\gamma ^2} \end{smallmatrix}}\right] \right) , \; B = I, \; C = I, \; D = 0, \end{aligned}$$

and nonlinear system $\mathscr {F}$ as in (3), setting

$$\begin{aligned} f(s, p) = (-\textrm{diag}(\beta ^1 s) W +\textrm{diag}(\gamma ^1))p^1 + (-\textrm{diag}(\beta ^2 s) W +\textrm{diag}(\gamma ^2))p^2, \end{aligned}$$

with $M_1 = I$, $$M_2 = \left[ {\begin{smallmatrix} {\textrm{diag}(\beta ^1) W} & {0} \\ {0} & {\textrm{diag}(\beta ^2) W} \end{smallmatrix}}\right]$$. The set of initial conditions of interest is

$$\begin{aligned} \mathscr {I} := \{(s, x) : s \gg 0, p_1 +p_2>0; s + p_1 + p_2= 1_{n_s}\}. \end{aligned}$$

The bi-virus nSIS model can then be expressed as $[G, \mathscr {F}]$. It can be verified that $M_2CA^{-1}BM_1$ is irreducible. Suppose for all $(s(0),x(0))\in \mathscr {I}$, $s(t)\rightarrow \bar{s}$ and $x(t)\rightarrow \bar{x}$, then by Theorem 1, it holds for some $i\in \{1,\ldots , n_s\}$ that

$$\begin{aligned} \bar{s}_i\le&\frac{1}{\max \{\rho (\textrm{diag}(\beta ^1) W\textrm{diag}(\frac{1}{\gamma ^1}), \rho (\textrm{diag}(\beta ^2) W\textrm{diag}(\frac{1}{\gamma ^2})\}} =\frac{1}{\max \{\rho (W\textrm{diag}(\frac{\beta ^1}{\gamma ^1})), \rho (W\textrm{diag}(\frac{\beta ^2}{\gamma ^2}))\}} =: \frac{1}{R_0}. \end{aligned}$$

The assumption on the convergence of $(s(t),p^1(t),p^2(t))$ is ensured under appropriate conditions^34^. Specifically, if

6 $$\begin{aligned} \lambda (-\textrm{diag}(\gamma ^j)+\textrm{diag}(\beta ^j)W) \cap \mathbb {C}_+ \ne \emptyset \end{aligned}$$

7 $$\begin{aligned} \lambda (-\textrm{diag}(\gamma ^k)+\textrm{diag}(\beta ^k)W) \cap \bar{\mathbb {C}}_+ = \emptyset , \end{aligned}$$

where $j, k \in \{1, 2\}$ and $j \ne k$, it can be concluded by applying Ref. [34, Theorem 2] that for all initial conditions $(s(0),p_j(0),p_k(0))\in \{s\ge 0, p^j>0, p^k\ge 0, s+p^i+p^j = 1_{n_s}\}\supseteq \mathscr {I}$, the system converges to a unique equilibrium $(\bar{s},\bar{p}_1,\bar{p}_2)$ with $\bar{p}_j\gg 0$ and $\bar{p}_k=0$. Note that (6) is equivalent to $\rho (W\textrm{diag}(\frac{\beta ^j}{\gamma ^j}))> 1$ and (7) to $\rho (W\textrm{diag}(\frac{\beta ^k}{\gamma ^k})) \le 1$.

##### Example 2

**Fig. 4:**
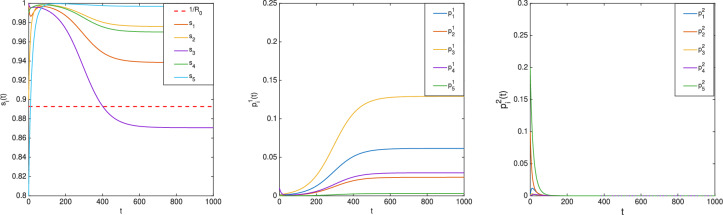
Evolution of $(s(t),p^1(t),p^2(t))$ in the bi-virus nSIS model (the first case).

Consider a bi-virus nSIS model over the network depicted in Fig. 2. Let $\beta ^1_1=0.2,\beta ^1_2=\beta ^1_3=\beta ^1_4=0.1,\beta ^1_5=0.05$, $\beta ^2_1=\beta ^2_3=\beta ^2_4=\beta ^2_5=0.1,\beta ^2_2=0.2$ and $\gamma ^1_1=0.3,\gamma ^1_2=\gamma ^1_4=0.2,\gamma ^1_3=\gamma ^1_5=0.1$, $\gamma ^2_1=\gamma ^2_4=0.2,\gamma ^2_2=\gamma ^2_3=0.3,\gamma ^2_5=0.15$, which satisfy (6) and (7) with $j=1$. Thus, $(s(t),p^1(t),p^2(t))$ converges to $(\bar{s}, \bar{p}^1, 0)$ for all initial conditions in $\mathscr {I}$ by Ref. [34, Theorem 2]. According to the preceding result, there exists *i* such that $\bar{s}_i<\frac{1}{R_0}=0.8926$. Let the initial condition be chosen from $\mathscr {I}$ as $s(0)=[1\; 0.9\; 1\;0.09\;0.8]^T, p^1(0)=[0\; 0\; 0\;0.01\;0]^T, p^2(0)=[0\; 0.1\; 0\;0\;0.2]^T$, which simulates the scenario that virus 1 spreads from Node 4 and virus 2 originates from Nodes 2 and 5. The evolution of $(s(t),p^1(t),p^2(t))$ is plotted in Fig. 4, where it can be seen that $\bar{s}_3 < \frac{1}{R_0}$.

**Fig. 5:**
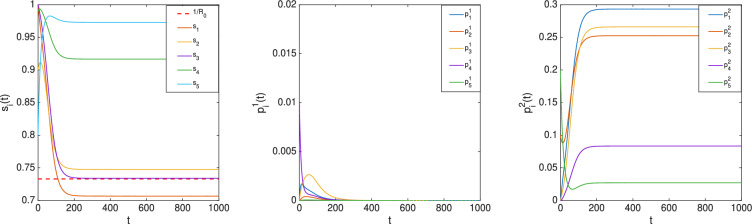
Evolution of $(s(t),p^1(t),p^2(t))$ in the bi-virus nSIS model (the second case).

Consider the same example but with different recovery rates for virus 2 given by $\gamma ^2_1=\gamma ^2_3=0.1,\gamma ^2_2=\gamma ^2_4=0.2,\gamma ^2_5=0.15$, which satisfy (6) with $j=2$. With the same initial condition as above, the evolution of $(s(t),p^1(t),p^2(t))$ is shown in Fig. 5, where it can be seen that $\bar{s}_1<\frac{1}{R_0}=0.733$.

For the simplicity of illustration, the graph *W* was taken to be the same for both the viruses. It should be noted that Theorem 1 is applicable to the case with different contact graphs — one for each virus.

### Networked-SIR models

Let $s_i(t)$, $p_i(t)$, and $r_i(t)$ denote the proportions of population that are susceptible, infected, and recovered, respectively, at time *t* and node *i*. The nSIR model^9^ is described by

$$\begin{aligned} \dot{s}_i(t)&= -\beta _i s_i(t) \sum _{j = 1}^N w_{ij} p_j(t) \\ \dot{p}_i(t)&= \beta _i s_i(t) \sum _{j = 1}^N w_{ij} p_j(t) - \gamma _i p_i(t) \\ \dot{r}_i(t)&= \gamma _i p_i(t), \end{aligned}$$

where $\beta _i$ and $\gamma _i$ denote the infection and recovery rates at node *i*, respectively. Writing this in vectorial form $s = [s_i]$, $p = [p_i]$, $r = [r_i]$, $\beta = [\beta _i]$, and $\gamma = [\gamma _i]$ yields

$$\begin{aligned} \dot{s}(t)&= -\textrm{diag}(s(t)\beta ) W p(t) \\ \dot{p}(t)&= \textrm{diag}(s(t)\beta ) W p(t) - \textrm{diag}(\gamma ) p(t) \\ \dot{r}(t)&= \textrm{diag}(\gamma ) p(t). \end{aligned}$$

Define LTI system *G* as in (1) by setting

$$\begin{aligned} x(t) = p(t), \; A = - \textrm{diag}(\gamma ), \; B = I, \; C = I, \; D = 0, \end{aligned}$$

and nonlinear component $\mathscr {F}$ as in (3), setting

$$\begin{aligned} f(s, v) = -\textrm{diag}(\beta s) W v, \end{aligned}$$

with $M_1 = I$, $M_2 = \textrm{diag}(\beta ) W$. The set of initial conditions of interest is

$$\begin{aligned} \mathscr {I} := \{(s, p) : 0\le s \ll 1_{n_s}, p> 0; s + p \le 1_{n_s}\}. \end{aligned}$$

The nSIR model can thus be expressed as $[G, \mathscr {F}]$.

Observe that each entry in *s*(*t*) is monotonically nonincreasing and bounded from below, so it converges by the *monotone convergence theorem*. Let $\bar{s}:=\lim _{t \rightarrow \infty } s(t)$, then Theorem 2 says that there exists *i* such that

$$\begin{aligned} \bar{s}_i < \frac{1}{\rho (M_2\hat{G}(0))} = \frac{1}{\rho (W\textrm{diag}(\frac{\beta }{\gamma }))} =: \frac{1}{R_0}. \end{aligned}$$

As explained previously, the bound is especially informative if $R_0> 1$, in which case $\bar{s}_i \le \frac{1}{R_0} < 1$. In particular, the higher the value of $R_0$, the lower the upper bound of $\bar{s}_i$, which means that the disease would penetrate more deeply into at least one of the population groups and at least $1 - \frac{1}{R_0}$ of the population in that group would have been infected. Theorem 3 may be used to compute $\bar{s}$ and $\lim _{t \rightarrow \infty } r(t)$.

#### Example 3

**Fig. 6:**
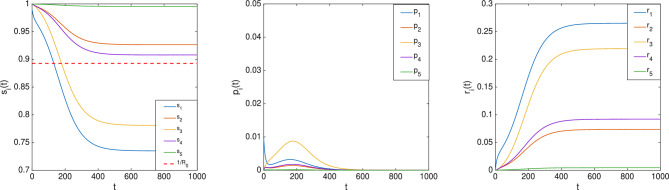
Evolution of $(s_i(t),p_i(t), r_i(t)), i=1,\ldots ,5$ in the nSIR model.

Consider an nSIR model over a network depicted in Fig. 2. Let $\beta _1=0.2,\beta _2=\beta _3=\beta _4=0.1,\beta _5=0.05$ and $\gamma _1=0.3,\gamma _2=\gamma _4=0.2,\gamma _3=\gamma _5=0.1$. It follows that $R_0=1.1203>1$. Therefore, according to the preceding result, there exists *i* such that $\bar{s}_i<\frac{1}{R_0}=0.8926$. Let the initial condition be $s(0)=[0.99\; 1\; 1\;1\;1\;]^T, p(0)=[0.01\; 0\; 0\;0\;0\;]^T, r(0)=[0\;0\;0\;0\;0]^T,$ which simulates the scenario that the spread starts from Node 1. The simulation plot is illustrated in Fig. 6, where it can be seen that $\bar{s}_1$ and $\bar{s}_3$ are less than $\frac{1}{R_0}$. Additionally, as predicted by Theorem 2, $\lim _{t\rightarrow \infty }p(t)=0$.

The nSIR model with vital dynamics is given by

$$\begin{aligned} \dot{s}(t)&= \mu - \textrm{diag}(\mu )s(t) - \textrm{diag}(s(t)\beta ) W p(t) \\ \dot{p}(t)&= \textrm{diag}(s(t)\beta ) W p(t) - \textrm{diag}(\gamma ) p(t) - \textrm{diag}(\mu ) p(t) \\ \dot{r}(t)&= \textrm{diag}(\gamma ) p(t) - \textrm{diag}(\mu ) r(t), \end{aligned}$$

where $\mu = [\mu _i]$ and at each node *i*, there is an inflow of newborns into the susceptible compartment $s_i$ at rate $\mu _i> 0$ and deaths in all the compartments at rates $\mu _i s_i$, $\mu _i p_i$, and $\mu _i r_i$, respectively.

**Fig. 7:**
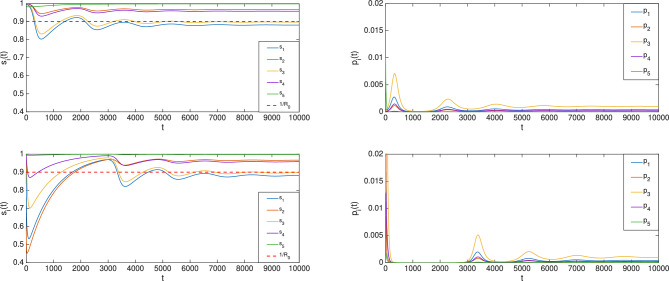
Evolution of $(s_i(t),p_i(t)), i=1,\ldots ,5$ in the nSIR model with vital dynamics. The top two panels correspond to the initial condition $s(0) = [1\;1\;1\;1\;0.99]^T$, $p(0) = [0\;0\;0\;0\;0.01]^T$, $r(0) = 0$, while the bottom two panels correspond to $s(0) = [0.9\;0.6\;1\;1\;1]^T$, $p(0) = [0.1\;0.4\;0\;0\;0]^T$, $r(0) = 0$.

Define LTI system *G* as in (1) by setting

$$\begin{aligned} x(t) = p(t), \, A = - \textrm{diag}(\gamma + \mu ), \, B = \textrm{diag}(\beta ), \, C = I, \, D = 0, \end{aligned}$$

and nonlinear component $\mathscr {F}$ as in (3), setting

$$\begin{aligned} f(s, v) = \mu -\textrm{diag}(\mu )s - \textrm{diag}(\beta s) W v, \end{aligned}$$

with $M_1 = I$, $M_2 = W$. It can be verified that $M_2CA^{-1}BM_1$ is irreducible. The set of initial conditions of interest is

$$\begin{aligned} \mathscr {I} := \{(s, p) : s \gg 0, p> 0; s + p \le 1_{n_s}\}. \end{aligned}$$

Suppose, as a standing assumption, that $s(t) \rightarrow \bar{s}$ and $x(t) \rightarrow \bar{x}$ for all $(s(0), x(0)) \in \mathscr {I}$, then Theorem 1 says that there exists *i* such that

$$\begin{aligned} \bar{s}_i \le \frac{1}{\rho (M_2\hat{G}(0))} = \frac{1}{\rho (W\textrm{diag}(\frac{\beta }{\gamma +\mu }))} =: \frac{1}{R_0}. \end{aligned}$$

#### Example 4

Consider again Example 3 but with vital dynamics and let $\mu =0.001\cdot [1\;1\;1\;1\;1]^T$. It follows that $R_0=1.1109>1$. With the initial condition chosen as $s(0)=[1\;1\;1\;1\;0.99]^T, p(0)=[0\;0\;0\;0\;0.01]^T, r(0)=0$, the evolution of (*s*(*t*), *p*(*t*)) is shown in the top two panels in Fig. 7. In contrast, the bottom two panels of Fig. 7 illustrate the evolution of (*s*(*t*), *p*(*t*)) when the initial condition is chosen differently, as $s(0)=[0.9\;0.6\;1\;1\;1]^T, p(0)=[0.1\;0.4\;0\;0\;0]^T, r(0)=0$. As can be seen in Fig. 7, the steady-state values for both cases are the same and $\bar{s}_1 < \frac{1}{R_0}$.

Building upon the nSIR model, the nSIRS model is described by

$$\begin{aligned} \dot{s}(t)&= - \textrm{diag}(s(t)\beta ) W p(t) + \textrm{diag}(\delta ) r(t) \\ \dot{p}(t)&= \textrm{diag}(s(t)\beta ) W p(t) - \textrm{diag}(\gamma ) p(t) \\ \dot{r}(t)&= \textrm{diag}(\gamma ) p(t) - \textrm{diag}(\delta ) r(t), \end{aligned}$$

where $\delta =[\delta _i]$ with $\delta _i$ being the rate at which immunity recedes following recovery at node *i*. Define LTI system *G* as in (1) by setting

$$\begin{aligned} x(t)=\left[ {\begin{smallmatrix} {p(t)} \\ {r(t)} \end{smallmatrix}}\right] , \; A = \left[ {\begin{smallmatrix} {-\textrm{diag}(\gamma )} & {0} \\ {\textrm{diag}(\gamma )} & {-\textrm{diag}(\delta )} \end{smallmatrix}}\right] , \; B=\left[ {\begin{smallmatrix} {I} \\ {0} \end{smallmatrix}}\right] , \; C=I, \end{aligned}$$

$D=0$ and the nonlinear component $\mathscr {F}$ as in (3), setting

$$\begin{aligned} f(s,v)= \left[ {\begin{smallmatrix} {-\textrm{diag}(s\beta )W \;}&{\textrm{diag}(\delta )} \end{smallmatrix}}\right] v, \end{aligned}$$

with $M_1 = I$, $$M_2 = \left[ {\begin{smallmatrix} {\textrm{diag}(\beta )W\;}&{0} \end{smallmatrix}}\right]$$. It can be verified that $M_2CA^{-1}BM_1$ is irreducible. Consider the set of initial conditions of interest

$$\begin{aligned} \mathscr {I} := \{(s, p, r) : s \gg 0, p> 0, r \ge 0; s + p + r = 1_{n_s}\}. \end{aligned}$$

Suppose, as a standing assumption, that $s(t)\rightarrow \bar{s}$ and $x(t)\rightarrow \bar{x}$ for all $(s(0,x(0)))\in \mathscr {I}$. Theorem 1 then states that there exists $i\in \{1,\ldots ,n_s\}$ such that $\bar{s}_i \le \frac{1}{R_0}$, where $R_0:= \rho (M_2\hat{G}(0)) = \rho (W \textrm{diag}(\frac{\beta }{\gamma }))$.

### Networked-SEIR models

Let $s_i(t)$, $e_i(t)$, $p_i(t)$, and $r_i(t)$ denote the proportions of population that are susceptible, exposed, infected, and recovered, respectively, at time *t* and at node $i \in \{1, \ldots , N\}$. The networked-SEIR (nSEIR) model Ref. [3, Section 3.3] is described by

$$\begin{aligned} \dot{s}(t)&= - [\textrm{diag}(s(t)\beta _{E}) W e(t) +\textrm{diag}(s(t)\beta _{I}) W p(t)] \\ \dot{e}(t)&= [\textrm{diag}(s(t)\beta _{E}) W e(t) + \textrm{diag}(s(t)\beta _{I}) W p(t)] - \textrm{diag}(\sigma ) e(t) \\ \dot{p}(t)&= \textrm{diag}(\sigma ) e(t) - \textrm{diag}(\gamma ) p(t) \\ \dot{r}(t)&= \textrm{diag}(\gamma ) p(t), \end{aligned}$$

where $\sigma =[\sigma _i]$, $\beta _E=[\beta _{Ei}]$, $\beta _I = [\beta _{Ii}]$ and at node *i*, $e_i$ denotes the proportion of the population that has been exposed to the disease, $\sigma _i$ the transition rate from exposed to infected, $\beta _{Ei}$ and $\beta _{Ii}$ represent the transmission rates between susceptible and exposed, and susceptible and infected, respectively.

Define LTI system *G* as in (1) by setting

$$\begin{aligned} x(t) = \left[ {\begin{smallmatrix} {e(t)} \\ {p(t)} \end{smallmatrix}}\right] , \; A = \left[ {\begin{smallmatrix} {-\textrm{diag}(\sigma )} & {0} \\ {\textrm{diag}(\sigma )} & {-\textrm{diag}(\gamma )} \end{smallmatrix}}\right] , \; B = \left[ {\begin{smallmatrix} {I} \\ {0} \end{smallmatrix}}\right] , \; C = I, \end{aligned}$$

$D = 0$ and nonlinear component $\mathscr {F}$ as in (3), setting

$$\begin{aligned} f(s, v) = -\left[ {\begin{smallmatrix} {\textrm{diag}(s\beta _{E})W\;}&{\textrm{diag}(s\beta _{I})W} \end{smallmatrix}}\right] v, \end{aligned}$$

with $M_1 = I$, $$M_2 = \left[ {\begin{smallmatrix} {\textrm{diag}(\beta _E)W\;}&{\textrm{diag}(\beta _I)W} \end{smallmatrix}}\right]$$. The set of initial conditions of interest is

$$\begin{aligned} \mathscr {I} := \{(s, e, p) : 0\le s \ll 1_{n_s}, \; e \ge 0, \; p \ge 0, e+p>0, \; s + p + e \le 1_{n_s}\}. \end{aligned}$$

Observe that each entry in *s*(*t*) is monotonically nonincreasing and bounded from below, so it converges by the *monotone convergence theorem*. Suppose $s(t) \rightarrow \bar{s}\gg 0$, then Theorem 2 says that there exists *i* such that

$$\begin{aligned} \bar{s}_i&< \frac{1}{\rho (M_2\hat{G}(0))} = \frac{1}{\rho (\textrm{diag}(\beta _E)W\textrm{diag}(\sigma )^{-1} + \textrm{diag}(\beta _I)W\textrm{diag}(\gamma )^{-1})} =: \frac{1}{R_0}. \end{aligned}$$

Theorem 3 is applicable for evaluating $\bar{s}$, $\lim _{t \rightarrow \infty } p(t)$ and $\lim _{t \rightarrow \infty } e(t)$.

#### Example 5

Consider an nSEIR model over a network of 5 nodes depicted in Fig. 2 with $\beta _E = [0.2\;0.1\;0.1\;0.1\;0.05]^T, \beta _I = 0.2\cdot [1\;1\;1\;1\;1]^T, \sigma = 0.1\cdot [1\;1\;1\;1\;1]^T , \gamma = [0.3\;0.2\;0.1\;0.2\;0.1]^T$. It follows that $R_0=3.6987>1$. Suppose the epidemic is caused by a tiny minority of the population in Node 5 having been exposed to the disease, as captured by the initial condition

8 $$\begin{aligned} \begin{aligned} s(0)=[1\;1\;1\;1\;0.9999]^T ,\quad e(0)=[0\;0\;0\;0\;0.0001]^T ,\quad p(0)=r(0)=0. \end{aligned} \end{aligned}$$

**Fig. 8:**
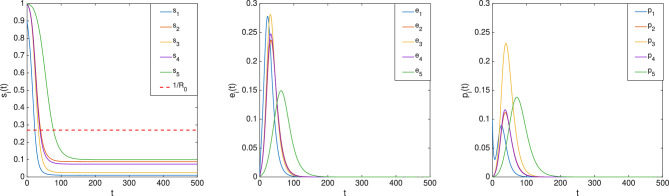
Evolution of $(s_i(t) e_i(t),p_i(t)),{i=1,\ldots ,5}$ in the nSEIR model.

The evolution of (*s*(*t*), *e*(*t*), *p*(*t*)) is shown in Fig. 8. It can be seen that $\displaystyle \lim _{t\rightarrow \infty }e(t)=\displaystyle \lim _{t\rightarrow \infty }p(t)=0$, and every $s_i(t)$ converges to a value less than $\frac{1}{R_0}$.

The nSEIR model with vital dynamics is described by

$$\begin{aligned} \dot{s}(t)&= \mu - \textrm{diag}(\mu )(s(t)) - \textrm{diag}(s(t)\beta _{E}) W e(t) - \textrm{diag}(s(t)\beta _{I}) W p(t) \\ \dot{e}(t)&= \textrm{diag}(s(t)\beta _{E}) W e(t) + \textrm{diag}(s(t)\beta _{I}) W p(t) - \textrm{diag}(\sigma ) e(t) - \textrm{diag}(\mu )e(t)\\ \dot{p}(t)&= \textrm{diag}(\sigma ) e(t) - \textrm{diag}(\gamma ) p(t) - \textrm{diag}(\mu ) p(t) \\ \dot{r}(t)&= \textrm{diag}(\gamma ) p(t) - \textrm{diag}(\mu ) r(t). \end{aligned}$$

Define LTI system *G* as in (1) by setting

$$\begin{aligned} x(t) = \left[ {\begin{smallmatrix} {e(t)} \\ {p(t)} \end{smallmatrix}}\right] , A = \left[ {\begin{smallmatrix} {-\textrm{diag}(\sigma + \mu )} & {0} \\ {\textrm{diag}(\sigma )\;} & {-\textrm{diag}(\gamma + \mu )} \end{smallmatrix}}\right] , B = \left[ {\begin{smallmatrix} {I} \\ {0} \end{smallmatrix}}\right] , C = I, \end{aligned}$$

$D = 0$ and nonlinear component $\mathscr {F}$ as in (3), setting

$$\begin{aligned} f(s, v) = \mu - \textrm{diag}(\mu )s -\left[ {\begin{smallmatrix} {\textrm{diag}(s\beta _{E})W\;}&{\textrm{diag}(s\beta _{I})W} \end{smallmatrix}}\right] v, \end{aligned}$$

with $M_1 = I$, $$M_2 = \left[ {\begin{smallmatrix} {\textrm{diag}(\beta _E)W}&{\textrm{diag}(\beta _I)W} \end{smallmatrix}}\right]$$. The set of initial conditions of interest is

$$\begin{aligned} \mathscr {I} := \{(s, e, p) : s \gg 0, e \ge 0, p \ge 0, e+p>0, s + p + e \ll 1_{n_s}\}. \end{aligned}$$

Suppose, as a standing assumption, that $s(t) \rightarrow \bar{s}$ and $x(t)\rightarrow \bar{x}$ for all $(s(0), x(0)) \in \mathscr {I}$, then Theorem 1 says that there exists *i* such that $\bar{s}_i \le \frac{1}{\rho (M_2\hat{G}(0))}= \frac{1}{R_0}$, where

$$\begin{aligned} R_0&:= \rho \Bigg (\textrm{diag}(\beta _E)W\textrm{diag}(\sigma + \mu )^{-1} + \textrm{diag}(\beta _I)W\textrm{diag}\left( \frac{\sigma }{(\sigma +\mu )(\gamma +\mu )}\right) \Bigg ). \end{aligned}$$

**Fig. 9:**
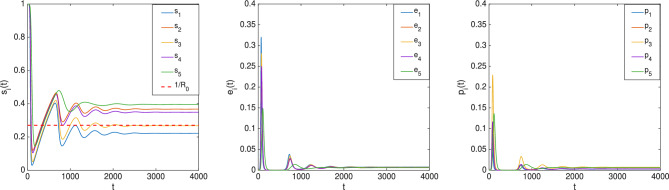
Evolution of $(s_i(t),e_i(t),p_i(t)), {i=1,\ldots ,5}$ in the nSEIR models with vital dynamics.

#### Example 6

Consider again the nSEIR model introduced in Example 5, but appended with vital dynamics specified by rate $\mu =0.001\cdot [1\;1\;1\;1\;1]^T$. It follows that $R_0=3.6482>1$. Let the initial condition be given by (8). The evolution of (*s*(*t*), *e*(*t*), *p*(*t*)) is shown in Fig. 9. In particular, observe that $\bar{s}_1<\frac{1}{R_0}$.

## Conclusion

We developed a positive-feedback framework for the steady-state analysis of epidemic models and demonstrated that the reciprocal of the basic reproduction number (BRN) quantifies the extent of disease penetration into at least one subgroup within a networked epidemic model. Two markedly different scenarios were analyzed: one in which an endemic state exists, and another where it does not. These cases were shown to exhibit distinct dynamic behaviors in the positive feedback system.

In the absence of an endemic state, we derived explicit formulas for computing convergence limits in the epidemic models. Various illustrative examples across different compartmental models were studied and simulated to validate and highlight our findings.

Future research should extend this framework to discrete-time models^39^ and incorporate control strategies in epidemic modeling^2,10,40,41^, with a focus on quantifying efficiency and expected penetration levels. To this end, it is crucial to investigate how changes in network topology^42,43^ and variations in infection or recovery rates influence BRNs–and consequently, the expected disease penetration. Such changes may result from public health interventions, including quarantine, isolation, social distancing, mask mandates, or vaccination programs.

## Supplementary Information

Supplementary Information.

## Data Availability

The code supporting the findings of this study is available from the corresponding author upon reasonable request.
