# Supplementary material for: Long-term susceptible fractions in networked epidemic models and their relation to the basic reproduction number
Source: Sci Rep. 2025 Nov 3;15:38429. doi: 10.1038/s41598-025-22158-0 (PMC12583584; doi:10.1038/s41598-025-22158-0)
Supplement: Supplementary file 1 — Supplementary Information. [file 41598_2025_22158_MOESM1_ESM.pdf]

Supplementary Information for *Long-Term Susceptible Fractions in Networked Epidemic Models and Their Relation to the Basic Reproduction Number*

Sei Zhen Khong[1], Lanlan Su[2,*], and Tryphon T. Georgiou[3]

[1] Department of Electrical Engineering, National Sun Yat-sen University, Kaohsiung 804201, Taiwan.
[2]Department of Electrical and Electronic Engineering, University of Manchester, Manchester, M13 9PL, UK.
[3]Department of Mechanical and Aerospace Engineering, University of California, Irvine, CA 92697, USA.
[*]Corresponding email: lanlan.su@manchester.ac.uk

This document provides detailed mathematical proofs of the main technical results presented in the main article. The supplementary material is self-contained and includes restatements of key lemmas and theorems for clarity.

The following result from [1, Corollary 2.1.5] is important for subsequent developments.

Lemma 1. (i) *If* $0 \leq M \leq N$, *then* $\rho(M) \leq \rho(N)$; (ii) *If* $0 \leq M < N$ *and* $M + N$ *is irreducible, then* $\rho(M) < \rho(N)$.

Lemma 2. *Consider an internally positive LTI system described by*

$$\begin{aligned} \dot{x}(t) &= Ax(t) + Bu(t), \quad x(0) = x_0 \\ y(t) &= Cx(t) + Du(t), \end{aligned} \tag{1}$$

with Hurwitz A *and* $D = 0$. *Given* $K \geq 0$, *it holds that* $(I - K\hat{G})^{-1} \in \mathbf{RH}_\infty$ *if and only if* $\rho(K\hat{G}(0)) < 1$.

Proof. Sufficiency follows from [2, Theorem 3(i)]. For necessity, note that $(I - KG)^{-1} = w \mapsto u$ may be described by the LTI state-space model

$$\begin{aligned} \dot{x}(t) &= (A + BKC)x(t) + Bw(t) \\ u(t) &= KCx(t) + w(t). \end{aligned}$$

Observe that this system is internally positive. Suppose to the contrapositive that $\rho(K\hat{G}(0)) \geq 1$. By [2, Theorem 3(ii) and (iii)], this then implies that $(I - K\hat{G})^{-1} \notin \mathbf{RH}_\infty$. □

Theorem 1. *Consider the feedback system* $[G, \mathcal{F}]$ *described by* (1),

$$\mathcal{F}: \quad \begin{cases} \dot{s}(t) = f(s(t), v(t)), \quad s(0) = s_0 \\ z(t) = M_1 \left(I_d \otimes \operatorname{diag}(s(t))\right) M_2 v(t), \end{cases} \tag{2}$$

and

$$v = y, \quad u = z, \tag{3}$$

with A *being Hurwitz and* $D = 0$. *Suppose* $M_2 C A^{-1} B M_1$ *is irreducible, and that for all initial conditions* $(s(0), x(0)) \in \mathscr{I}$, $\lim_{t\to\infty} s(t) = \bar{s} \gg 0$ *and* $\lim_{t\to\infty} x(t) = \bar{x} \geq 0$. *Then there exists* $i \in \{1, \ldots, n_s\}$ *such that*

$$\bar{s}_i \leq \frac{1}{\rho(M_2 \hat{G}(0) M_1)} = \frac{1}{\rho(M_2 C A^{-1} B M_1)}.$$

Moreover, if $n_s = 1$, *then* $\bar{s} = \frac{1}{\rho(M_2 \hat{G}(0) M_1)}$.

Proof. Since $s(t) \to \bar{s}$, it follows that for sufficiently large $T > 0$, by approximating $\mathcal{F}$ by the constant gain

$$\tilde{\mathcal{F}} = v \mapsto z : z(t) = M_1 \left(I_d \otimes \operatorname{diag}(\bar{s})\right) M_2 v(t) =: K v(t)$$

for $t \geq T$, the closed-loop system $[G, \tilde{\mathcal{F}}]$ described by

$$\dot{x}(t) = (A + BKC) x(t) \tag{4}$$

is a close approximation of the dynamics in $[G, \mathcal{F}]$ for $t \geq T$. The fact that $x(t) \to \bar{x} \geq 0$ then implies that $\lambda(A + BKC) \subset \bar{\mathbb{C}}_-$, as established below.

Suppose to the contrapositive that $\lambda(A + BKC) \cap \mathbb{C}_+ \neq \emptyset$. Since $A + BKC$ is Metzler, we can write it as $A + BKC = M - bI$ for some $b > 0$ and $M \geq 0$. By the Krein-Rutman theorem for nonnegative matrices [1, Theorem 2.1.1], there then exists $\delta x > 0$ such that

$$(A + BKC)\delta x = (M - bI)\delta x = \kappa\, \delta x$$

for some $\kappa > 0$. Note that δx may be chosen to be sufficiently small. Since $\bar{s} \gg 0$, there exists $\delta s > 0$ such that $(\bar{s} - \delta s, \bar{x} + \delta x) \in \mathscr{I}$ and $|\delta s| = |\delta x|$. Setting

$$(s(0), x(0)) = (\bar{s} - \delta s, \bar{x} + \delta x) \in \mathscr{I}$$

in (1), (2), and (3) then yields in the steady-state approximation (4) that $|x(t)| \to \infty$, leading to a contradiction to $x(t) \to \bar{x}$. Therefore, it must hold that $\lambda(A + BKC) \subset \bar{\mathbb{C}}_-$, which implies $\lambda(A - \alpha I + BKC) \subset \mathbb{C}_-$ for all $\alpha > 0$. Now consider

$$\begin{aligned} \dot{x}(t) &= (A - \alpha I + BKC) x(t) + B w(t) \\ u(t) &= KC x(t) + w(t), \end{aligned}$$

which describes the closed-loop system

$$(I - \tilde{\mathcal{F}} G_\alpha)^{-1} = w \mapsto u,$$

where $\hat{G}_\alpha(s) := C(sI-(A-\alpha I))^{-1}B$. Evidently, the LTI system above is internally positive. Since $A - \alpha I + BKC$ is Hurwitz, it follows that $(I - \tilde{\mathcal{F}}\hat{G}_\alpha(s))^{-1} \in \mathbf{RH}_\infty$. From Lemma 2, $(I - \tilde{\mathcal{F}}\hat{G}_\alpha(s))^{-1} \in \mathbf{RH}_\infty$ if and only if

$$\rho(\tilde{\mathcal{F}}\hat{G}_\alpha(0)) < 1. \tag{5}$$

By continuity, as $\alpha \to 0$, we have $\rho(\tilde{\mathcal{F}}\hat{G}(0)) \leq 1$, where $\hat{G}(s) = C(sI - A)^{-1}B$.

Recall from [1, Theorem 6.2.3] [3, Proposition 1] that the Metzler matrix A is Hurwitz if and only if $-A^{-1} \geq 0$. Thus, $M_2CA^{-1}BM_1 \geq 0$. Observe that

$$\rho(\tilde{\mathcal{F}}\hat{G}(0)) = \rho(-M_1 \left(I_d \otimes \operatorname{diag}(\bar{s})\right) M_2CA^{-1}B) = \rho(-\left(I_d \otimes \operatorname{diag}(\bar{s})\right) M_2CA^{-1}BM_1). \tag{6}$$

If $\bar{s} = \frac{1}{\rho(M_2CA^{-1}BM_1)} 1_{n_s}$, then clearly $\rho(\tilde{\mathcal{F}}\hat{G}(0)) = 1$. By hypothesis, $-M_2CA^{-1}BM_1 \geq 0$ is irreducible, and it follows that $-M_2CA^{-1}BM_1 > 0$ and $\rho(M_2CA^{-1}BM_1) > 0$.

Now let $M = (I_d \otimes \operatorname{diag}(\bar{s}))\, M_2CA^{-1}BM_1$ where $\bar{s} = \frac{1}{\rho(M_2CA^{-1}BM_1)} 1_{n_s}$ and $N = (I_d \otimes \operatorname{diag}(\bar{s}))$ $M_2CA^{-1}BM_1$ with $\bar{s} > \frac{1}{\rho(M_2CA^{-1}BM_1)} 1_{n_s}$. Note that $0 \leq M < N$, and that both M and N are irreducible, so is their sum. Therefore, by Lemma 1, $\rho(N) > \rho(M) = 1$. That means, if $\bar{s} > \frac{1}{\rho(M_2CA^{-1}BM_1)} 1_{n_s}$, then $\rho(\tilde{\mathcal{F}}\hat{G}(0)) > 1$. In other words, $\rho(\tilde{\mathcal{F}}\hat{G}(0)) \leq 1$ implies that there exists i such that $\bar{s}_i \leq \frac{1}{\rho(M_2CA^{-1}BM_1)}$. This completes the proof for the first claim.

By the same reasoning leading to $\bar{s} > \frac{1}{\rho(M_2CA^{-1}BM_1)} 1_{n_s}$ implies $\rho(\tilde{\mathcal{F}}\hat{G}(0)) > 1$ above, it may be shown similarly that $\bar{s} < \frac{1}{\rho(M_2CA^{-1}BM_1)} 1_{n_s}$ implies $\rho(\tilde{\mathcal{F}}\hat{G}(0)) < 1$. Therefore, since $\rho(\tilde{\mathcal{F}}\hat{G}(0)) = 1$, it holds that there exists i such that $\bar{s}_i < \frac{1}{\rho(M_2CA^{-1}BM_1)}$ if and only if there exists $j \neq i$ such that $\bar{s}_j > \frac{1}{\rho(M_2CA^{-1}BM_1)}$. The claim on $n_s = 1$ then follows. □

Assumption 1. *If $\bar{s} > 0$ and $f(\bar{s}, \bar{v}) = 0$, then $\bar{v} = 0$.*

Theorem 2. *Consider the feedback system $[G, \mathcal{F}]$ described by (1), (2), and (3) with A being Hurwitz and $D = 0$. Suppose that Assumption 1 holds and that for all initial conditions $(s(0), x(0)) \in \mathscr{I}$, $\bar{s}(s(0), x(0)) := \lim_{t\to\infty} s(t)$ exists and is continuous in $x(0)$. Then, $\lim_{t\to\infty} x(t) = 0$, and*

$$\bar{s}_i(s(0), x(0)) < \frac{1}{\rho(M_2\hat{G}(0)M_1)} = \frac{1}{\rho(M_2CA^{-1}BM_1)} \tag{7}$$

for some $i \in \{1, \ldots, n_s\}$.

Proof. Given $(s(0), x(0)) \in \mathscr{I}$, since $\bar{s}(s(0), x(0))$ exists, it must hold that

$$f(\bar{s}(s(0), x(0)), v(t)) = f(\bar{s}(s(0), x(0)), y(t)) \to 0$$

as $t \to \infty$. Moreover, if $\bar{s}(s(0), x(0)) > 0$, then this implies by Assumption 1 that $y(t) \to 0$. Since (C, A) is detectable, it follows that $x(t) \to 0$. For sufficiently large $T > 0$, by approximating $\mathcal{F}$ by the constant gain

$$\tilde{\mathcal{F}} = v \mapsto z : z(t) = M_1 \operatorname{diag}(\bar{s}(s(0), x(0))) M_2 v(t) =: Kv(t)$$

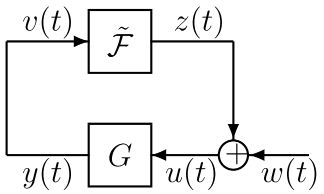

Figure 1: The feedback system $[G, \tilde{\mathcal{F}}]$

for $t \geq T$, the closed-loop system $[G, \tilde{\mathcal{F}}]$ described by

$$\dot{x}(t) = (A + BKC)x(t) \tag{8}$$

is a close approximation of the dynamics in $[G, \mathcal{F}]$ for $t \geq T$. Note that if $\bar{s}(s(0), x(0)) = 0$, then trivially $K = 0$ and $A + BKC = A$ is Hurwitz, in which case $x(t) \to 0$.

Consider $1_n \gg \bar{s}(s(0), x(0)) > 0$. Since $A + BKC$ is Metzler, the fact that $x(t) \to 0$ then implies that $A + BKC$ is Hurwitz. To see this, suppose $\lambda(A + BKC) \cap \bar{\mathbb{C}}_+ \neq \emptyset$. Write $A + BKC = M - bI$ for some $b > 0$ and $M \geq 0$. By the Krein-Rutman theorem for nonnegative matrices [1, Theorem 2.1.1], there then exists $\delta x > 0$ such that

$$(A + BKC)\delta x = (M - bI)\delta x = \kappa\, \delta x$$

for some $\kappa \geq 0$. Setting

$$(s(0), x(0)) = (\bar{s} + \tau 1_{n_s}, \delta x) \in \mathscr{I}$$

in (1), (2), (3) for sufficiently small $\tau > 0$ and $\delta x > 0$ for which $\tau \gg |\delta x|$, and exploiting the continuity of $\bar{s}$ in $x(0)$ then yields in the steady-state approximation (8) a perturbed K, called $\tilde{K}$, so that $\bar{K} \geq K$ and $|K - \tilde{K}| \to 0$ as $\tau \to 0$ and $|\delta x| \to 0$. It follows from Lemma 1 that $\lambda(A + B\tilde{K}C) \cap \bar{\mathbb{C}}_+ \neq \emptyset$, and hence

$$\dot{x}(t) = (A + BKC)x(t) \approx (A + B\tilde{K}C)x(t) \approx \kappa x(t)$$

satisfies either $\lim_{t\to\infty} x(t)$ is nonzero or $|x(t)| \to \infty$. This leads to a contradiction to $x(t) \to 0$. As such, $A + BKC$ must be Hurwitz, whereby $x(t) \to 0$. Now, define

$$\begin{aligned} \dot{x}(t) &= (A + BKC)x(t) + Bw(t) \\ u(t) &= KCx(t) + w(t), \end{aligned} \tag{9}$$

which is internally positive and describes the closed-loop system in Fig. 1, i.e., $(I - \tilde{\mathcal{F}}G)^{-1} = w \mapsto u$.

Hurwitzness of $A + BKC$ implies that $(I - \tilde{\mathcal{F}}\hat{G})^{-1} \in \mathbf{RH}_\infty$, where $\hat{G}(s) = C(sI - A)^{-1}B$. By Lemma 2, $(I - \tilde{\mathcal{F}}\hat{G})^{-1} \in \mathbf{RH}_\infty$ if and only if $\rho(\tilde{\mathcal{F}}\hat{G}(0)) < 1$. Recalling (6), if $\bar{s} = \frac{1}{\rho(M_2CA^{-1}BM_1)} 1_{n_s}$, then clearly $\rho(\tilde{\mathcal{F}}\hat{G}(0)) = 1$. Furthermore, by Lemma 1, if $\bar{s} \geq \frac{1}{\rho(M_2CA^{-1}BM_1)} 1_{n_s}$, then $\rho(\tilde{\mathcal{F}}\hat{G}(0)) \geq 1$. Hence, $\rho(\tilde{\mathcal{F}}\hat{G}(0)) < 1$ implies that there exists i such that $\bar{s}_i < \frac{1}{\rho(M_2CA^{-1}BM_1)}$. □

Theorem 3. *Consider the feedback system* $[G, \mathcal{F}]$ *described by* (1), (2), *and* (3) *with* $d = 1$, A *being Hurwitz and* $D = 0$. *Suppose* (i) *Assumption 1 holds,* (ii) $M_1 = I$, $f(s, v) = -\mathrm{diag}(s)M_2v$, *and* (iii) *for all* $(s(0), x(0)) \in \mathscr{I}$ *such that* $s(0) \gg 0$, *it holds that* $s(t) \gg 0, \forall t \geq 0$ *and* $\lim_{t\to\infty} s(t) = \bar{s} \gg 0$. *Then* $\bar{s}$ *satisfies*

$$\log\left(\mathrm{diag}(s(0))^{-1}\bar{s}\right) + M_2CA^{-1}B(\bar{s} - s(0)) = M_2CA^{-1}x(0).$$

Proof. First, by the same arguments as in the proof of Theorem 2, the existence of $\lim_{t\to\infty} s(t)$ implies that $x(t) \to 0$. By hypothesis,

$$\dot{s}(t) = -\mathrm{diag}(s(t))M_2v(t),$$

whereby $\frac{d}{dt}\log(s(t)) = \mathrm{diag}(s(t))^{-1}\dot{s}(t) = -M_2v(t)$.

Since $u(t) = z(t) = \mathrm{diag}(s(t))M_2v(t)$, it follows that $u(t) = -\dot{s}(t)$. From (1), (2), and (3), we have

$$\begin{aligned}\int_0^\infty \dot{x}(t)\,dt = x(\infty) - x(0) = -x(0) &= A\int_0^\infty x(t)\,dt + B\int_0^\infty u(t)\,dt \\ &= A\int_0^\infty x(t)\,dt - B\int_0^\infty \dot{s}(t)\,dt.\end{aligned}$$

Premultiplying the equation above by $-M_2CA^{-1}$ yields

$$\begin{aligned}M_2CA^{-1}x(0) &= -M_2\int_0^\infty v(t)dt + M_2CA^{-1}B\int_0^\infty \dot{s}(t)dt \\ &= \int_0^\infty \frac{d}{dt}\log(s(t))dt + M_2CA^{-1}B\int_0^\infty \dot{s}(t)dt \\ &= \log\left(\mathrm{diag}(s(0))^{-1}\bar{s}\right) + M_2CA^{-1}B(\bar{s} - s(0)),\end{aligned}$$

as required.

□

References

[1] A. Berman and R.J. Plemmons. *Nonnegative matrices in the mathematical sciences.* Society for Industrial and Applied Mathematics, 1994.

[2] T. Tanaka, C. Langbort, and V. Ugrinovskii. DC-dominant property of cone-preserving transfer functions. *Systems and Control Letters*, 62:699–707, 2013.

[3] A. Rantzer. Scalable control of positive systems. *European Journal of Control*, 24:72–80, 2015.
